# Bacterial Infection and Implant Loosening in Hip and Knee Arthroplasty: Evaluation of 209 Cases

**DOI:** 10.3390/ma9110871

**Published:** 2016-10-26

**Authors:** Ulrike Dapunt, Stephanie Radzuweit-Mihaljevic, Burkhard Lehner, Gertrud Maria Haensch, Volker Ewerbeck

**Affiliations:** 1 Center for Orthopaedics, Trauma Surgery and Spinal Cord Injury, Heidelberg University Hospital, Schlierbacher Landstrasse 200a, Heidelberg 69118, Germany; stephanie.radzuweit@me.com (S.R.-M.); Burkhard.Lehner@med.uni-heidelberg.de (B.L.); Sandra.Brix@med.uni-heidelberg.de (V.E.); 2 Institute for Immunology, Heidelberg University, Im Neuenheimer Feld 305, Heidelberg 69120, Germany; maria.haensch@urz.uni-heidelberg.de

**Keywords:** coagulase-negative *Staphylococcus* spp., hip arthroplasty, implant loosening, knee arthroplasty, prosthetic joint infection

## Abstract

The aim of this study was to evaluate bacteria species detected in a large number of patients treated for prosthetic joint infection of the hip and knee at a single specialized center. Furthermore, the rate of implant loosening was investigated in a time-dependent manner for the most frequently detected bacteria species. A retrospective analysis of patients (*n* = 209) treated for prosthetic joint infection of the hip and knee was performed. The following parameters were evaluated: C-Reactive Protein (CRP) concentration, microbiological evaluation of tissue samples, loosening of the implant, the time that had elapsed since the primary prosthetic joint replacement, and the duration since the last surgical intervention. Coagulase-negative *Staphylococcus* spp. were most frequently detected, followed by *Staphylococcus aureus.* Differences in CRP concentration were detected among various bacteria species. Osteolysis was not associated with one causative agent in particular. Patients who had undergone previous revision surgery had a higher probability of implant loosening. Coagulase-negative *Staphylococcus* spp. are the most common causative agents of prosthetic joint infection and show no significant differences with regard to implant loosening or the time-course when compared to *S. aureus.* Infections with *Enterococcus* spp. seem to develop faster than with other bacteria species. The risk of implant loosening increases with revision surgery, in particular in the hip joint.

## 1. Introduction

Total joint replacement is considered one of the most successful surgical procedures in the field of orthopaedics, alleviating pain and limitation of movement due to degenerative or traumatic joint damage. Despite this achievement, prosthetic joint infections still pose a severe complication, often leading to catastrophic results and requiring repeated and extensive treatment [1].

Infections of foreign bodies are particularly difficult to treat because bacteria protect themselves by forming sessile communities on the implant surface and embed themselves in a slimy matrix, the so called “biofilm” [2]. *Staphylococcus* spp. are considered the predominant bacteria associated with implant infections and *Staphylococcus aureus* is often thought to be the main causative agent [3], but also other bacteria species, coagulase-negative staphylococci in particular, have gained increasing attention. Coagulase-negative staphylococci have been described as the most common bacteria in early (within three months after surgery), delayed (three to 24 months after surgery) and late (>2 years after surgery) prosthetic joint infections [4,5]. *S. aureus* is often considered to be most likely in acute haematogenous infections but Gram-negative bacteria have also been described to play an important role in these infections [6]. Moreover, other Gram-positive bacteria (such as *Enterococcus* spp.) have also been described in association with implant infections and even an increase over the last 30 years has been proposed [6,7]. Therefore, the first aim of this study was the evaluation of bacteria species detected in a large number of patients treated for prosthetic joint infection (PJI) of the hip and knee at a single specialized center.

Additionally, we were able to demonstrate in our previous work that biofilms are recognized and attacked by neutrophils [8,9]. However, in some cases, effective clearance of the biofilm fails, which presumably results in a persistent inflammatory response, leading to osteoclast generation, osteolysis and hence implant loosening [10,11,12], the latter consequently necessitating implant-exchange surgery. Osteolysis is typically thought to be associated with delayed, low-grade infections predominantly caused by coagulase-negative staphylococci and loosening of an implant is a crucial factor for deciding on a surgical course of action [13,14].

Therefore, another aim of this study was to evaluate the rate of osteolysis occurring in hip and knee prosthetic joint infection and to analyze a possible time-dependence in association with microbiological results or systemic signs of an infection.

## 2. Patients and Methods

Patients (*n* = 209) treated surgically for a prosthetic joint infection (PJI) of the knee or hip at the University Hospital Heidelberg between January 2010 until December 2013 were evaluated retrospectively. Prosthetic joint infection was diagnosed according to the following criteria: clinical signs of an infection (reddening, swelling, hyperthermia, pain, pus intraoperative, fistula), laboratory diagnostics (elevated CRP concentration and white blood cell count), detection of bacteria by culture of tissue samples (one to four samples) or by joint aspiration and positive histological evaluation of tissue samples (>23 neutrophils per 10 high power fields) [15].

For tissue culture, samples were processed according to the following protocol. After arrival at the lab, the tissue was ground using a porcelain mortar, followed by the addition of 1 mL of 0.9% NaCl. This suspension was inoculated onto Columbia 5% sheep blood agar (BD), chocolate agar, MacConkey agar, SCS agar, Schaedler Neo Vanco +5% sheep blood (SNVS) agar (all BioMérieux, Marcy, France), and thioglycolate broth (BD), and then Gram staining was performed. Plates and broth were incubated until positive or for a maximum of five days at 36 °C in 5% CO_2_ or under anaerobic conditions. Identification of bacteria was done with the MALDI-TOF mass spectrometer. Susceptibility testing was done using the Vitek 2 microbial identification system (BioMérieux, Marcy, France).

The following parameters were evaluated in this study: Type of prosthesis (knee, hip), C-reactive Protein (CRP)-concentration (>5 mg/L), microbiological evaluation of tissue samples, loosening of the implant (osteolysis detected on X-ray, CT-scan or intraoperative documented loosening of the implant), the time that had elapsed since the primary prosthetic joint replacement measured in months, and the duration since the last surgical intervention of the prosthetic joint in question (in months).

Differences between groups were calculated using *t*-test, chi-square-test and Mann-Whitney test, respectively, using Origin 9.0 software (OriginLab, Northampton, MA, USA). Significance level was determined as *p* < 0.05.

## 3. Results

### 3.1. Clinical Data

Clinical data of patients included in this study are listed in Table 1.

Information regarding implant loosening could not be acquired retrospectively in 16 cases, duration since primary prosthetic joint replacement in six cases and duration since last surgical intervention in 13 cases.

### 3.2. Bacteria Species in Infected Hip and Knee Prostheses

In 159 of the 209 patients, bacteria were detected. Twenty of these patients had a polymicrobial infection (17 were double positive, two triple positive, and in one patient, four different types of bacteria were diagnosed). A multitude of bacteria were found (see Table S1), and only the five most frequent species were further investigated in this study. These were *Staphylococcus epidermidis* (occurring in 29.2% of the patients), *Staphylococcus aureus* (23.8%), other coagulase-negative *Staphylococcus* spp. (in 11.1%), *Enterococcus* spp. (in 10.5%), and *Streptococcus* spp. (in 8.8%).

**Figure 1:**
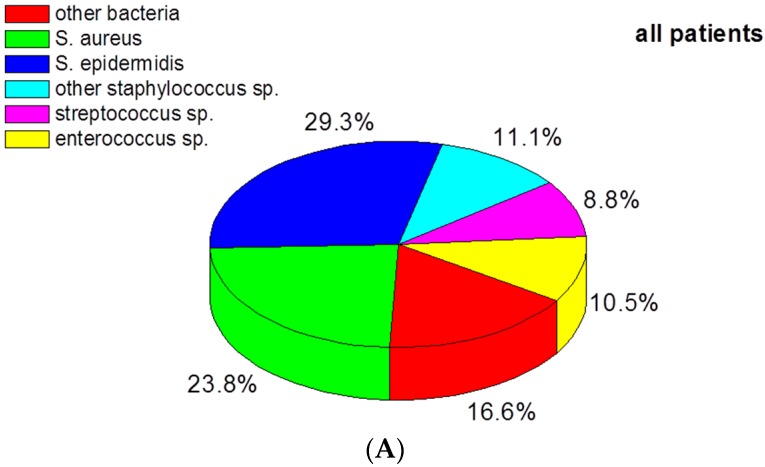

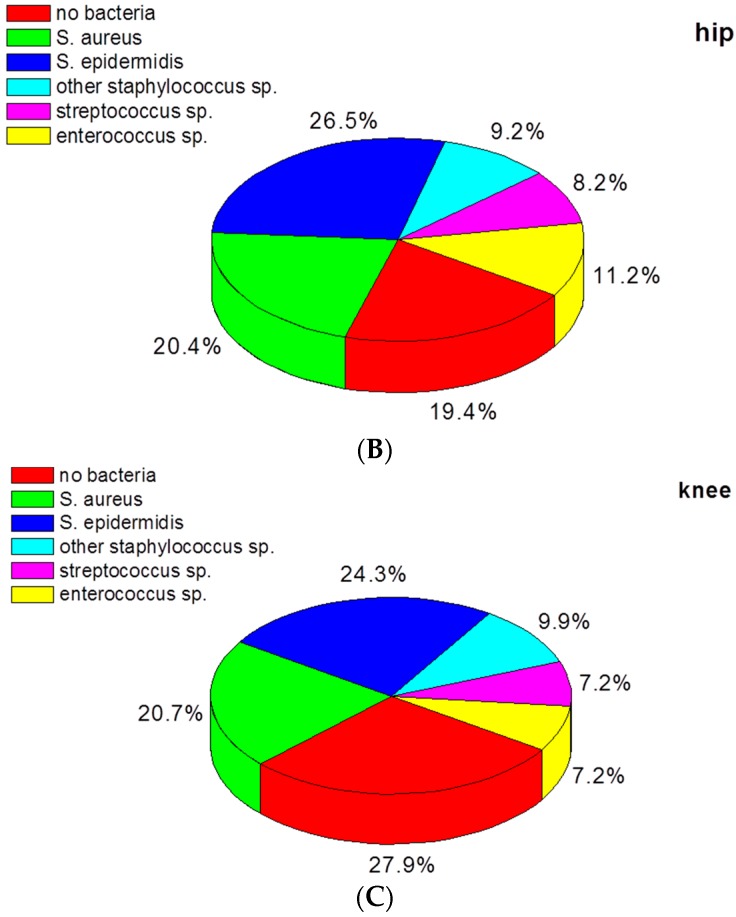
(**A**) Distribution of bacteria (*n* = 159) detected in all patients; (**B**,**C**) Numbers of culture-negative cases and of the five most frequently detected bacteria in prosthetic joint infection of the hip (**B**) and knee (**C**). Numbers were calculated proportional to the number of patients (*n* = 98, *n* = 111). Since some infections were polymicrobial and in some cases “other bacteria” were detected (which are not depicted in this graph), the total sum of the percentages displayed cannot amount to 100%.

The distribution of the bacteria species is depicted in Figure 1A. Of note, in 50 of the 209 patients bacteria could not be detected despite clinical assessment of a PJI. When hip (*n* = 98) and knee (*n* = 111) prostheses were evaluated separately, an essentially similar distribution of bacteria was seen (Figure 1B,C). The number of bacteria culture–negative cases (31/111) in patients with knee prostheses appeared to be higher compared to patients with hip prostheses (19/98). The difference between the groups, however, was not quite statistically significant according to Fisher’s exact test.

### 3.3. Association of CRP Concentration with Infection

**Figure 2:**
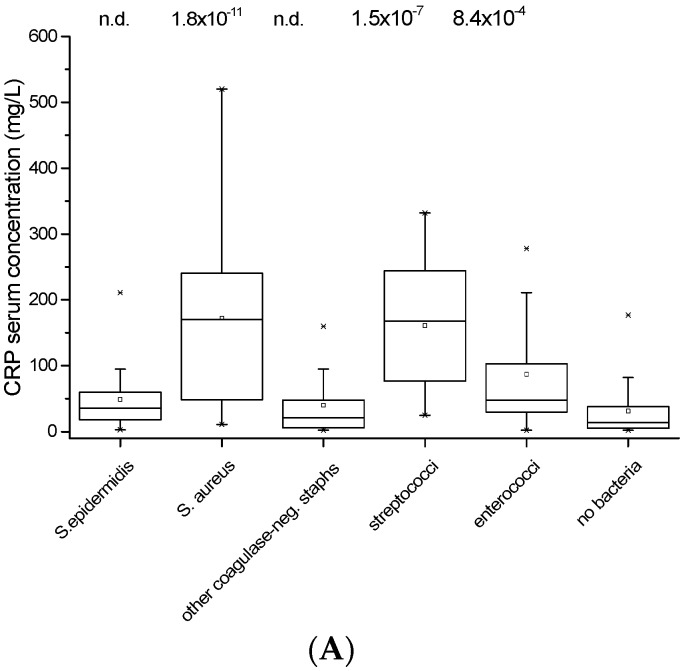

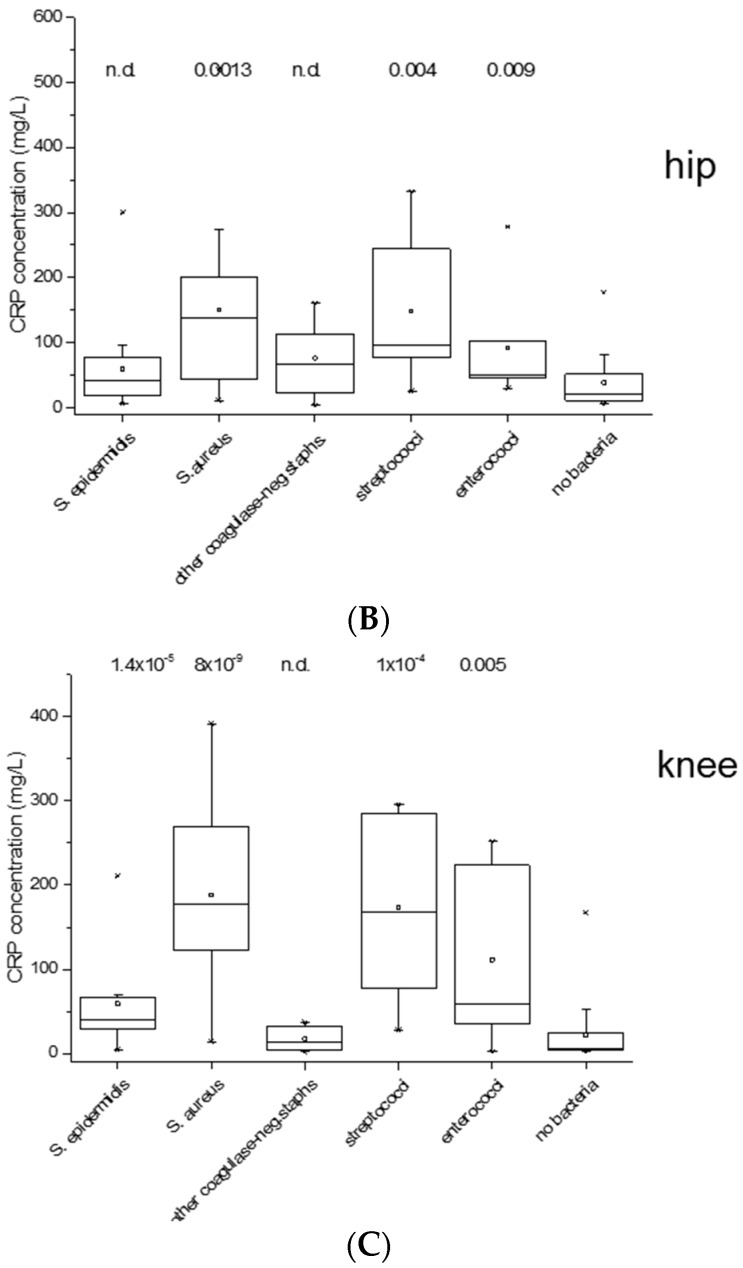
(**A**–**C**): CRP serum concentrations of all patients (**A**) included in the study, of patients with infected hip implants (**B**) and of patients with infected knee implants (**C**). Differences between culture-negative cases (no bacteria) and the five most frequent bacteria are calculated using the Mann-Whitney test. Significance level was determined as *p* < 0.05.

The CRP serum concentration was highest in patients with *S. aureus* infection or infection with *Streptococcus* spp., considerably lower in infection with the other bacteria (*S. epidermidis*, other *Staphylococcus* spp. and *Enterococcus* spp.), and lowest in patients who were culture-negative for bacteria (Figure 2A). Essentially similar results were obtained when patients with hip prostheses or knee prostheses were evaluated separately (Figure 2B,C).

### 3.4. Time Elapsed Since the Primary Prosthetic Joint Replacement and Since the Last Surgical Intervention

**Figure 3:**
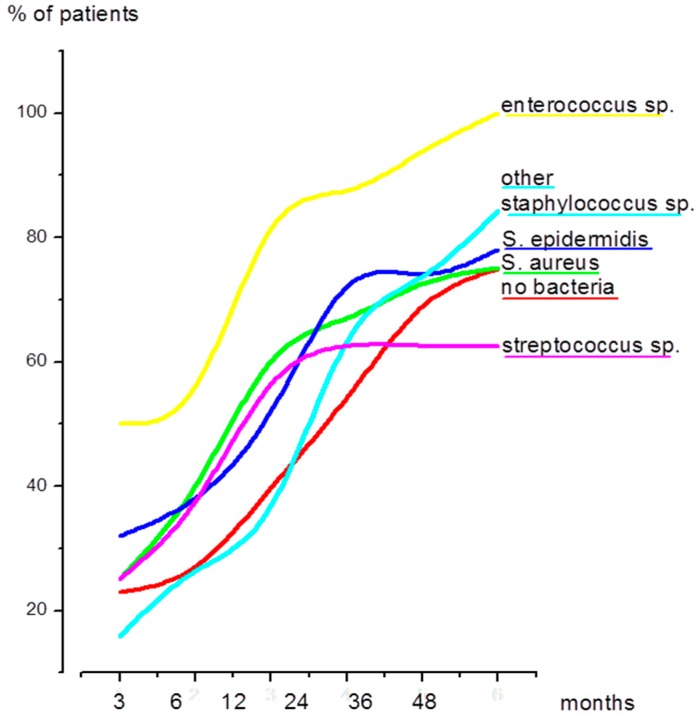
Percentage of patients treated after three, six, 12, 24, 36 and 48 months since their last surgical intervention of the prosthetic joint in question. Shown are the time-courses of the five most common bacteria species and culture-negative cases (no bacteria).

When all patients were considered, the median value since primary joint replacement was 38 months, but it varied slightly depending on the bacteria species. In patients with streptococci infection, there was a trend towards a prolonged duration compared to the median value; in patients with enterococci infection, there was a shorter time course (data summarized in Table 2). An essentially similar pattern was seen when the duration since the last surgical intervention was assessed, although the median of 12 months was considerably shorter (data summarized in Figure 3 and Table 2).

### 3.5. Implant Loosening in Hip and Knee Prostheses

**Figure 4:**
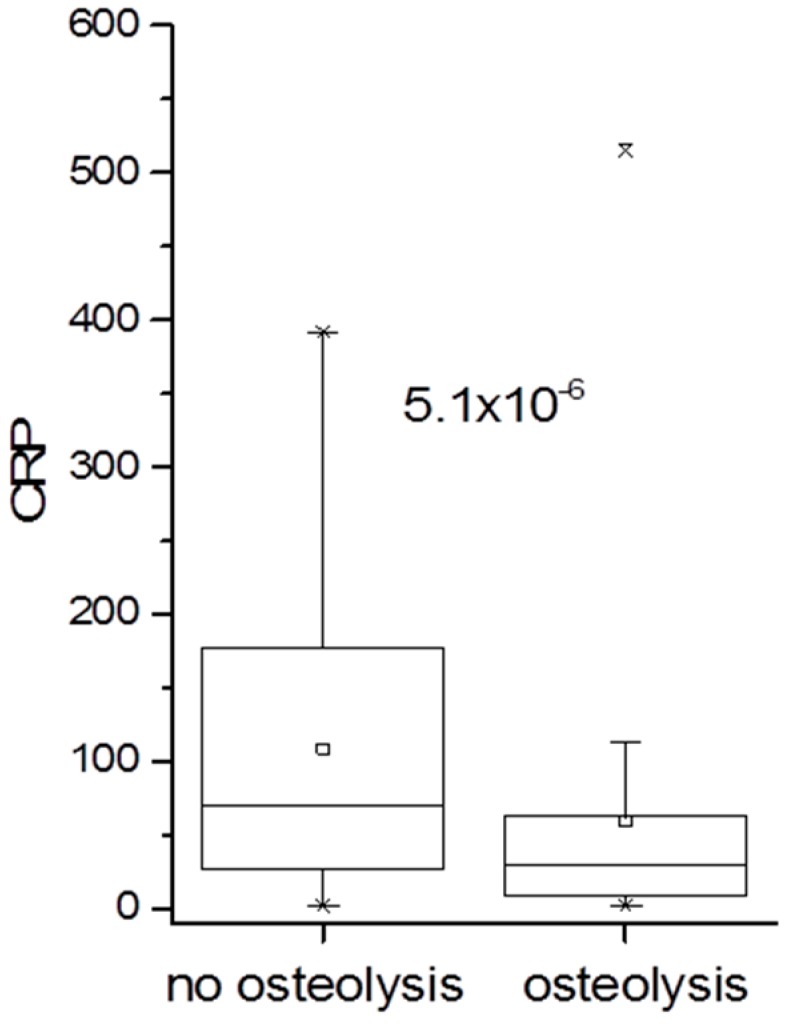
CRP serum concentration of patients with and without osteolysis. The difference was calculated using the Mann-Whitney test. Significance level was determined as *p* < 0.05.

A total of 100 (51.8%) patients showed signs of implant loosening: 55 patients with hip implants and 45 with knee implants. Of note, in patients with osteolysis the CRP concentration was significantly lower compared to that in patients without osteolysis (Figure 4).

Moreover, surgical intervention occurred earlier in patients without osteolysis compared to patients with osteolysis (median 11.5 months compared to 72 months when duration since the primary prosthetic joint replacement was considered, and 2.5 versus 36 months since the last surgical intervention; the differences are highly significant with *p* = 1.2 × 10^−9^ and *p* = 1.6 × 10^−13^, respectively). There was no conspicuous association between the bacteria species and the occurrence of osteolysis (data not shown).

We were also interested in comparing bacteria species detected and the rate of implant loosening in patients who were operated for the first time (*n* = 93) with patients who had undergone at least one previous revision surgery due to confirmed or suspected implant infection (*n* = 116). With regard to bacteria species, infection with *S. epidermidis* increased from 19.4% in patients with the first surgical procedure to 30.2% in patients with at least one previous revision surgery. Additionally, the rate of implant loosening was significantly higher in patients with prior revision surgery. For hips, the percentage of 44.7% increased to 75.4% in patients with at least one revision (*p* = 0.031), for knee prostheses from 30.0% to 53.4% (*p* = 0.026). Loosening of the prosthesis was generally more frequent in hip compared to knee implants.

## 4. Discussion

Prosthetic joint infections still pose one of the major complications in the field of orthopaedic surgery [5]. Infection rates are thought to be between 0.6% and 1.3% [16,17] for the hip joint and slightly higher (0.86%–2.5%) [4] for the knee joint, but considering the increasing number of total joint replacements performed each year—an estimated 328,112 joint replacements of the hip and knee were performed in Germany in 2013 [18]—even a small increase has detrimental effects.

The aim of this study was to retrospectively evaluate data of patients treated for prosthetic joint infection of the hip and knee at a single institution over the course of four years. During this time period, 209 patients were treated surgically due to PJI. Parameters of interest were bacteria species detected by culture of tissue samples, laboratory diagnostics indicative of infection (elevated CRP concentration), the time elapsed since primary joint replacement and the duration since the last surgical intervention, as well as evaluation of implant loosening.

Concerning bacteria species detected by culture of tissue samples, we found a similar distribution in hip and knee PJIs. *S. epidermidis* was overall the most frequently detected bacteria species and, taken together with other coagulase-negative *Staphylococcus* spp., this group even amounted to 40.3% and thus far outreached *S. aureus*, which was detected in 23.8% of the patients. This finding is in line with data by others and our results also support the notion that other Gram-positive bacteria, such as *Enterococcus* spp. (10.5%), have increased when compared to data from the late 1980s [6]. When we compared patients who were treated surgically due to PJI for the first time with patients who had already undergone at least one revision due to PJI, we found that *S. epidermidis* infections increased in the latter group. Contrary to data by others, Gram-negative bacteria were only rarely detected in our cases of prosthetic joint infection [19].

It has been argued that the CRP concentration can be misleading in diagnosing implant infection and extensive research is dedicated to finding a better systemic marker [20,21]. In our study 91% of cases that were clinically assessed as prosthetic joint infection did indeed show elevated CRP concentrations. These were highest in patients with *S. aureus*, *Strepotococcus* spp. or *Enterococcus* spp. infections, and significantly lower in infections with coagulase-negative *Staphylococcus* spp. or in patients who were culture-negative. The finding that *S. aureus* and *S. epidermidis*/other coagulase-negative *Staphylococcus* spp. induce a different systemic inflammatory response is in line with data in the literature [22,23,24] and indicates that the serum CRP concentration does not necessarily reflect the extent of local infection. This notion is supported by the observation that neither the time-course of infection nor osteolysis differ significantly between these species (discussed below).

Of particular interest is the time course of infection dependent on different bacteria species. Because the onset of an infection is usually not precisely traceable, we are well aware that the time points evaluated in this study are not impartial, but despite that shortcoming we were able to demonstrate that both *S. aureus* and *S. epidermidis* showed a similar time-course: about 70% of the patients were treated within two years since the last surgical intervention (see Figure 3). This finding is in contrast to data by others who showed that infections caused by *S. aureus* usually occur within 90 days [25]. Patients suffering from an *Enterococcus* spp. infection became symptomatic early on (50% within three months), even faster than previously described in the literature [26]. *Streptococcus* spp. Infections, on the other hand, developed later, which might be explained by a higher number of late haematogenous infections [6,27]. Of note, the group of other coagulase-negative *Staphylococcus* spp. showed the most delayed onset (also when compared to *S. epidermidis*) and, only after a duration of three years, the percentage of patients treated for *S. aureus*, *S. epidermidis* or other *Staphylococcus* spp. infections was similar.

The number of culture-negative cases in PJIs is controversially discussed in the literature and numbers span from 6% to 40% [6,14,28,29]. Our results demonstrate 27.9% of culture-negative cases in knee PJIs and 19.4% in hip PJIs despite clinical assessment of an infection. The question arises whether, in these patients, bacterial infection had escaped detection, or whether they were in fact aseptic. The low CRP concentration in these patients argues for the latter, however, as low CRP concentrations are also seen in patients with coagulase-negative *Staphylococcus* spp. infections. As mentioned before, the group of culture-negative cases showed a similar time-course as infections with other *Staphylococcus* spp. Moreover, we recently published our results on the sonication method in prosthetic joint infections [30]. Since bacteria are thought to be primarily attached to the implant surface, it has been shown that detection of bacteria can be enhanced by treating the implant with a mild ultrasound device (“sonication procedure”) [31,32,33,34,35]. We were able to demonstrate increased detection of bacteria and of coagulase-negative *Staphylococcus* spp. in particular by the sonication method, thus supporting the notion of undetected coagulase-negative *Staphylococcus* spp. infections in supposedly tissue culture–negative cases.

However, it is also possible that the high number of culture-negative cases might be due to the incubation period of tissue samples. Inoculated agar plates are usually incubated for five days to avoid secondary contamination and drying out of the plates. However, it has been argued to extend the incubation period to 14 days to improve detection of bacteria such as *Propionibacterium* spp. [5]. Furthermore, it has been proposed that numerous tissue specimens should be collected during surgery [5]. This was not always the case in the patients evaluated for this study. This aspect might also contribute to the high number of culture-negative cases.

With regard to implant loosening, 51.8% of all patients showed signs of osteolysis; 59.8% of hip prostheses were loose and 44.6% of knee prostheses. Implant loosening has been typically associated with delayed, low-grade infections. Therefore, we evaluated CRP concentrations in patients with and without osteolysis. CRP concentrations were indeed significantly lower in patients with osteolysis. The duration since primary prosthetic joint replacement and since the last surgical intervention was significantly longer in patients with implant loosening. However, as stated above, it is not possible to time osteolysis reliably since the onset of an infection cannot be established precisely. We used the day of surgery as an endpoint, but we acknowledge the fact that osteolysis has a protracted time course and that the need for surgery and hence the waiting period differs greatly with patients and external circumstances. Importantly, the occurrence of osteolysis was not related to the various bacteria species. Thus, we could not confirm the hypothesis that implant loosening is mainly associated with coagulase-negative *Staphylococcus* spp. Osteolysis, however, dramatically increased when patients had already undergone at least one previous surgical intervention of the affected prosthetic joint. In particular for hip joints, the number of loosened implants in this group increased to 75.6%.

In conclusion, we were able to demonstrate that coagulase-negative *Staphylococcus* spp. are the predominant causative agents of prosthetic joint infection. There were no significant differences between *S. epidermidis* and *S. aureus* with regard to implant loosening and time-course, despite the higher inflammation-inducing capacity of the latter. Certain bacteria species, *Enterococcus* spp. in particular, seem to be associated with an accelerated time-course. The rate of implant loosening is especially high in patients with previous revision surgery in general and of the hip joint specifically.

## Figures and Tables

**Table 1:** Summary of patients (*n* = 209) with prosthetic joint infection of the hip and knee included in the study; IQR (interquartile range).

Patients with Prosthetic Joint Infection	Hip	Knee
Number of patients	98	111
Age (years)	median 74	median 69
	range: 18–91	range: 27–91
	IQR: 16.5	IQR: 15
Gender	52 female	44 female
	46 male	67 male
elevated CRP serum concentration (reference value >5 mg/L)	98%	85%
Bacteria detected in tissue samples	80.60%	72.10%
Implant loosening	59.80%	44.6% *
Duration since primary prosthetic joint replacement (months)	median 48	median 36
	range: 1–408	range: 1–300
	IQR: 152	IQR: 75
Duration since last surgical intervention (months)	median 12	median 12
	range: 1–276	range: 1–180
	IQR: 47	IQR: 32

* value differs significantly as calculated by chi square (*p* = 0.04).

**Table 2:** Time (median value in months) elapsed since primary prosthetic joint replacement; IQR (interquartile range).

Bacteria Species	Hip	Knee
*S. aureus*	48	28
	(IQR 198)	(IQR 72.5)
*S. epidermidis*	41	36
	(IQR 129.5)	(IQR 109.5)
other *Staphylococcus* spp.	33	38
	(IQR 83)	(IQR 55)
*Streptococcus* spp.	162 *	61
	(IQR 197)	(IQR 64)
*Enterococcus* spp.	12 **	29
	(IQR 78)	(IQR 46)
no bacteria	72	54
	(IQR 154)	(IQR 84.5)

* significantly longer; ** significantly shorter compared to all staphylococci species.

## Supplementary Materials

The following are available online at www.mdpi.com/1996-1944/9/11/871/s1.
