# Supplementary material for: Bacterial Infection and Implant Loosening in Hip and Knee Arthroplasty: Evaluation of 209 Cases
Source: Materials (Basel). 2016 Oct 26;9(11):871. doi: 10.3390/ma9110871 (PMC5457256; doi:10.3390/ma9110871)
Supplement: Supplementary file 1 [file materials-09-00871-s001.pdf]

Supplementary Materials: Bacterial Infection and Implant Loosening in Hip and Knee Arthroplasty: Evaluation of 209 Cases

Ulrike Dapunt, Stephanie Radzuweit-Mihaljevic, Burkhard Lehner, Gertrud Maria Haensch and Volker Ewerbeck

Table S1. Causative agents detected in patients with prosthetic joint infection of the hip and knee.

Causative Agents Detected	Bacteria Species in Detail	Number
S. aureus	–	43
S. epidermidis		53
other staphylococcus species		20 (in detail)
	S. hominis,	4
	S. haemolyticus,	3
	S. caprae,	1
	S. lugdunensis,	5
	S. gallolyticus,	1
	S. capitis	6
Streptococcus sp.		16 (in detail)
	St. agalactiae,	1
	St. pneumoniae,	1
	St. parasanguinis,	1
	St. dysgalactiae,	2
	St. oralis,	4
	St. anginosus,	2
	St. canis,	1
	St. Group G,	1
	St. mitis,	1
	S. pyogenes	2
Enterococcus species	–	19
others		30 (in detail)
	Enterobacter cloacae,	3
	Parvimonas micra,	2
	Escherichia coli,	4
	Acinetobacter baumannii,	2
	Bacillus sp.,	3
	Pantoea sp.,	1
	Sphingobacterium multivorum,	1
	Finegoldia magna,	1
	Phialemonium sp.,	1
	Clostridium perfringens,	1
	Rothiadentocariosa,	1
	Serratia sp.,	1
	Actinomyces oris,	1
	Klebsiella pneumoniae,	2
	Pseudomonas aeruginosa,	2
	Micrococcus lentus,	1
	Proteus mirabilis,	2
	Propionibacterium sp.	1
